# The prevalence and long-term response to calcium channel blockers in patients with pulmonary arterial hypertension and positive vasoreactivity test – results of multicenter national registry (BNP-PL)

**DOI:** 10.1186/s13023-026-04250-4

**Published:** 2026-02-25

**Authors:** Michał Piłka, Szymon Darocha, Marcin Waligóra, Grzegorz Kopeć, Michał Florczyk, Adam Torbicki, Tatiana Mularek-Kubzdela, Anna Smukowska-Gorynia, Ewa Lewicka, Ewa Mroczek, Łukasz Chrzanowski, Piotr Błaszczak, Beata Kuśmierczyk, Katarzyna Ptaszyńska, Katarzyna Mizia-Stec, Ewa Malinowska, Małgorzata Peregud-Pogorzelska, Michał Tomaszewski, Wojciech Jacheć, Ilona Skoczylas, Zbigniew Gąsior, Agnieszka Pawlak, Miłosz Jaguszewski, Grzegorz Grześk, Katarzyna Betkier-Lipińska, Piotr Pruszczyk, Katarzyna Widejko, Judyta Winowska-Józwa, Marcin Kurzyna

**Affiliations:** 1 Chair and Department of Pulmonary Circulation, Thromboembolic Diseases and Cardiology, Center of Postgraduate Medical Education, European Health Center, ERN-LUNG Member, Borowa 14/18, Otwock, 05-400 Poland; 2 Department of Cardiac and Vascular Diseases, John Paul II Hospital in Krakow, Krakow, 31-202 Poland; 3 Pulmonary Circulation Centre, Department of Cardiac and Vascular Diseases, Faculty of Medicine, Jagiellonian University Medical College, Krakow, 31-008 Poland; 4 Center for Innovative Medical Education, Department of Medical Education, Faculty of Medicine, Jagiellonian University Medical College, Krakow, 30-688 Poland; 5 Department of Cardiology, Poznan University of Medical Sciences, Poznan, 61-701 Poland; 6 Department of Cardiology and Electrotherapy Medical University of Gdansk, Gdansk, 80-211 Poland; 7 Department of Cardiology, Institute of Heart Diseases, Wroclaw Medical University, Wroclaw, Poland; 8 Cardiology Department, Medical University of Lodz, Lodz, 91-347 Poland; 9 Department of Cardiology, Cardinal Wyszynski Hospital, Lublin, 20-718 Poland; 10 Department of Congenital Heart Disease Institute of Cardiology, Warsaw, 04-628 Poland; 11 Department of Cardiology, Medical University of Bialystok, Bialystok, 15-276 Poland; 12 First Department of Cardiology, School of Medicine in Katowice, Medical University of Silesia in Katowice, Katowice, 40-635 Poland; 13 Centre of the European Reference Network for Rare, Low Prevalence, or Complex Diseases of the Heart (ERN-GUARD Heart), Amsterdam, The Netherlands; 14 Pulmonary Department, University of Warmia and Mazury, Olsztyn, 10-357 Poland; 15 Department of Cardiology, Pomeranian Medical University, Szczecin, 70-111 Poland; 16 Department of Cardiology, Medical University of Lublin, Lublin, 20-090 Poland; 17 2nd Department of Cardiology, Faculty of Medical Sciences in Zabrze, Silesian Medical University in Katowice, Zabrze, 41-800 Poland; 18 3rd Department of Cardiology, Faculty of Medical Sciences in Zabrze, Medical University of Silesia, Katowice, Poland; 19 Department of Cardiology, School of Health Sciences in Katowice, Medical University of Silesia in Katowice, Katowice, 40-635 Poland; 20 Department of Invasive Cardiology, Polish Academy of Sciences, Mossakowski Medical Research Centre, Central Clinical Hospital of the Ministry of Interior, Warsaw, 02-507 Poland; 21 1st Department of Cardiology, Medical University of Gdańsk, Gdańsk, 80-210 Poland; 22 Department of Cardiology and Clinical Pharmacology, Collegium Medicum in Bydgoszcz, Nicolaus Copernicus University in Toruń, Toruń, Poland; 23 Department of Cardiology and Internal Medicine, Military Institute of Medicine - National Research Institute, Warsaw, 04-141 Poland; 24 Department of Internal Medicine and Cardiology with the Center for Diagnosis and Treatment of Venous Thromboembolism, Medical University of Warsaw, Warszawa, Poland; 25 Department of Cardiology, Copper Health Center, Lubin, 59-300 Poland; 26 Department of Cardiology, Provincial Specialist Hospital in Szczecin, Szczecin, Poland

**Keywords:** Pulmonary arterial hypertension, Calcium channel blockers, Acute pulmonary vasoreactivity test, Long-term responders, Non-long-term responders

## Abstract

**Background:**

The profile of patients with pulmonary arterial hypertension (PAH) and positive acute haemodynamic response (acute responders) who may benefit on long-term calcium channel blocker (CCB) therapy is not yet well documented.

**Results:**

The aim of this study was to identify the subgroup of patients with PAH from the Polish Multicenter Registry of Pulmonary Hypertension (BNP-PL) who were found to be responders at the initial acute pulmonary vasoreactivity test (APV-test), and analyse the frequency, characteristics and prognosis of patients with different long-term response to treatment with CCB. Out of 534 patients 49 (9.2%) fulfilled the responder criteria (acute responders). Patients with positive APV test had a less advanced WHO class (*p* < 0.001), longer distance in a 6-minute walking test (6MWT) and had a significantly lower NTproBNP compared to non-acute responders (540 m/IQR:480–560/ vs. 280 m/IQR:160–390/, *p* < 0.001; 503.2 pg/ml/IQR:214.6–1533/ vs. 2019 pg/ml/IQR:806–4286/, *p* < 0.001; respectively). The hemodynamic profile of the above two groups (acute responders and non-acute responders) was similar. CCB long-term response was found in 55.1% (*n* = 27) of acute responders. All-cause mortality was lower among acute responders (11.54%) compared to non-acute responders (36.07%); *p* = 0.039. Among acute responders, long-term responders showed markedly lower mortality (0%) compared to non-long-term responders (21.05%); *p* = 0.05.

**Conclusions:**

Novel analysis in a unique large Polish PAH population highlights that APV testing and rapid early post-CCB assessment are key to long-term prognosis.

## Background

Pulmonary Arterial Hypertension (PAH) is a rare disease characterized by remodelling and constriction of small pulmonary vessels, leading to increased pulmonary resistance (PVR) [1, 2]. In a small percentage of patients diagnosed with PAH, this is due to a dominant element is reversible constriction rather than progressive obliterative hypertrophy of pulmonary vessels [3]. This small group may be identified during the initial diagnostic right heart catheterization (RHC) by examining the reactivity of arterial vessels, including the assessment of changes in the hemodynamics of the arterial circulation [4]. The examination of arterial vascular reactivity is recommended in patients with idiopathic pulmonary arterial hypertension (IPAH), heritable pulmonary arterial hypertension (HPAH), and drug-induced pulmonary hypertension (DPAH) [2]. A positive acute pulmonary vasoreactivity test (APV-test) is helpful in selecting a group of patients who could benefit from treatment with high doses of calcium antagonists (so-called acute responders) [5–7]. At follow-up, some of these patients lose the ability to respond to treatment with high doses of calcium channel blockers (CCB) and require the introduction of additional specific treatment (non-long-term responders), while the others will maintain long-term positive response to the treatment with CCB (long-term responders) [8]. Although guidelines proposed by the European Society of Cardiology in 2022 provide a hemodynamic definition of long-term responder, there are currently scarce data in the literature on the prevalence and long-term follow-up, including response to treatment with CCB, of this very rare group of patients [2]. The aim of this study was to identify the subgroup of patients with PAH from the Polish multicenter registry of pulmonary hypertension (BNP-PL) who were found to be responders at the initial APV-test, and analyse the frequency, characteristics and prognosis of patients with different long-term response to treatment with CCB.

## Methods

### Study population

The study analysis included data of patients from the Database of Pulmonary Hypertension in the Polish population (BNP‑PL) of patients with PAH diagnosed between 1 March 2018 and 1 September 2023, who underwent an APV-test, i.e. patients with IPAH, DPAH, and HPAH [9, 10]. The data were collected by 21 reference centers of diagnosis and treatment of PH accredited by the National Health Fund. The APV test was conducted in accordance with previously established guidelines—recommended vasodilators for vasoreactivity testing include inhaled nitric oxide or iloprost. Evidence supporting intravenous epoprostenol is comparable; however, due to the need for gradual dose escalation and repeated measurements, the procedure is significantly more time-consuming and therefore performed less frequently [11]. The patients who met the hemodynamic criterion of responder received a pharmacotherapy with a CCB according to ESC/ERS guidelines [2, 12]. All the patients agreed to participate in the study. The study protocol conforms to the ethical guidelines of the Declaration of Helsinki. The Bioethics Committee approved the study protocol.

### Right heart catheterization (RHC) and acute pulmonary vasoreactivity test (APV-test)

PAH was confirmed by RHC performed by an experienced operator in accordance with standards [11]. Precapillary PH was defined in accordance with then applicable standards of the European Society of Cardiology, namely: mean pulmonary artery pressure (mPAP) ≥ 25 mmHg or mPAP > 20 mmHg, pulmonary vascular resistance (PVR) > 3 Wood units or PVR > 2 Wood units, pulmonary artery wedge pressure (PAWP) ≤ 15 mmHg; hemodynamic definition corresponding to standards applicable at the given time period [2, 12]. The cardiac output (CO) was calculated by thermodilution or Fick method. In addition, the following hemodynamic parameters were measured or calculated in the RHC: mPAP, mixed venous oxygen saturation (SvO₂), mean right atrial pressure (RAP). A positive APV-test was defined as a decrease in the mPAP value by at least 10 mmHg, and reaching values lower than 40 mmHg, without a decrease in CO [2, 12].

### Clinical assessment

The assessment of clinical parameters in the groups who underwent an APV-test was conducted. The following clinical data were assessed and compared: basic characteristics of the study population, WHO functional class, NTproBNP level, selected echocardiographic - ECHO (RVOT, TAPSE, RAA, pericardial effusion, pulmonary artery) and electrocardiographic - ECG (cardiac axis deviation, intraventricular conduction disorders) parameters, and 6MWT (6-minute walking test). ECHO and ECG was performed in accordance with applicable standards [13, 14]. 6MWT was conducted and interpreted in accordance with the guidelines of the American Thoracic Society [15].

The assessment of long-term response to treatment with a CCBs in the group of acute-responders (first clinical evaluation) was conducted in a median time of 10.7 (IQR:4.1–17.4) months from treatment initiation (one day after diagnostic RHC).

### Long-term responders to CCBs – definition

In this study, long-term responders were identified using a simplified clinical definition. Patients were classified as long-term responders if they were in WHO functional class I or II and remained on calcium channel blocker (CCB) monotherapy at the first clinical evaluation, whereas patients in WHO functional class III or IV, or those in whom CCB monotherapy was discontinued and additional PAH-specific therapy was initiated, were classified as non-long-term responders. In patients with a positive acute vasoreactivity test, high-dose CCB therapy (nifedipine, diltiazem, or amlodipine) was administered and titrated to the maximum tolerated dose in accordance with routine clinical practice. Detailed information on the specific type and dosing of CCB therapy during follow-up was not systematically captured, which may have resulted in heterogeneity of CCB exposure and should be considered when interpreting outcome differences between groups. This pragmatic definition was chosen to reflect real-world clinical practice; however, it differs from current ESC/ERS hemodynamic criteria and may be associated with a risk of misclassification. Survival in both groups was followed for up to 36 months.

### Statistical analysis

Continuous variables with normal distribution were described with the use of a mean and standard deviation, whereas continuous variables with a distribution other than normal were described with a median and interquartile range. The Shapiro-Wilk test was used to assess normality of the distribution of the analyzed variables. To compare variables in groups, depending on the distribution, a T-student test or Mann-Whitney test was used for continuous variables, and chi-square test or Fisher exact test was used for discrete variables. The Kaplan-Meier method was used to analyze survival, and the estimated survival curves were compared by means of a log-rank test. The significance level for the whole study was set at *p* < 0.05. Data analysis was performed with the use of Stata, 15.1 statistical software (StataCorp. 2017. Stata Statistical Software: Release 15. StataCorp LLC, College Station, TX, USA).

## Results

### Baseline characteristics of study population

**Fig. 1:**
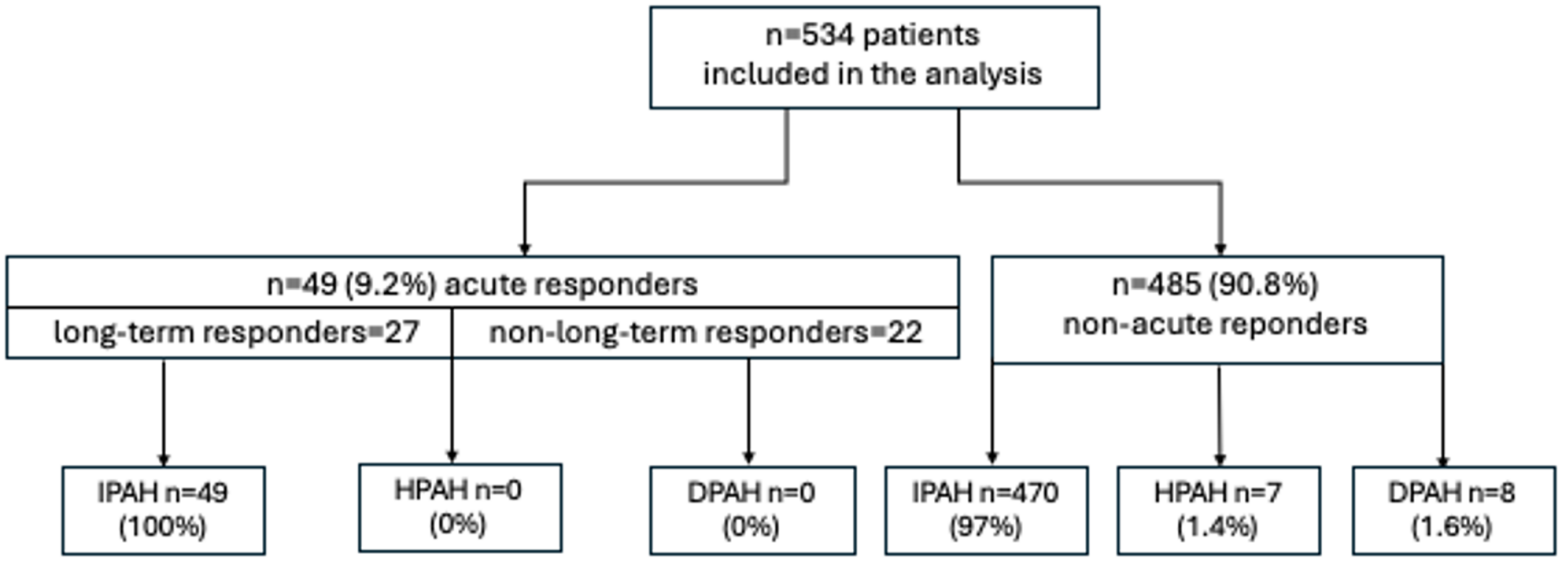
The flowchart showing characteristics of the study population. DPAH, drug-induced pulmonary hypertension; HPAH, heritable pulmonary arterial hypertension; IPAH, idiopathic pulmonary arterial hypertension

The total number of patients subject to an APV-test and included in the analysis was 534. The analysis was conducted on consecutive patients who correctly performed the above test. In the group of 534 patients included in the analysis, there were 519 (97.2%) patients with IPAH, 7 with HPAH (1.3%) and 8 with DPAH (1.5%). Out of 534 patients, 49 (9.2%) fulfilled the criteria of a positive APV-test (acute responders). All the patients in the group of acute responders were diagnosed with IPAH, none of them with HPAH or DPAH (Fig. 1).

Patients with a positive APV-test were younger than those with a negative test and had a lower prevalence of coexisting diseases (obesity, arterial hypertension, diabetes mellitus, chronic obstructive pulmonary disease/asthma, chronic renal failure, atrial fibrillation). Basic characteristics of the patients enrolled in the study are presented in Table 1.

**Table 1:** Basic characteristics of patients subject to an APV-test and included in the analysis (IPAH/ HPAH/ DPAH)

Characteristics	Acute responders *n* = 49	Non-acute responders *n* = 485	*p*-value
IPAH *n* = 519 (97.2%)	49 (100.0)	470 (97)	0.382
HPAH *n* = 7 (1.3%)	0 (0.0)	7 (1.4)	1.000
DPAH *n* = 8 (1.5%)	0 (0.0)	8 (1.6)	1.000
Age [y], median (IQR)	51 (31–65)	68 (52–74)	**< 0.001**
Sex, n (%)			
Female	35 (71.4)	292 (60.2)	0.124
Male	14 (28.6)	193 (39.8)	
BMI [kg/m²], median (IQR)	26.2 (22.2–29.7)	27.8 (24.0-32.1)	**0.046**
Arterial hypertension, n (%)	16 (32.7)	312 (64.3)	**< 0.001**
Diabetes mellitus, n (%)	8 (16.3)	179 (36.9)	**0.004**
Current smoking, n (%)	0 (0)	22 (4.5)	0.248
Coronary artery disease, n (%)	8 (16.3)	118 (24.3)	0.206
Chronic obstructive pulmonary disease/Asthma, n (%)	4 (8.2)	109 (22.5)	**0.017**
Hypothyroidism, n (%)	13 (26.5)	104 (21.4)	0.416
Chronic renal failure (eGFR < 60 ml/min/1.73 m²), n (%)	5 (10.2)	121 (24.9)	**0.020**
Atrial Fibrillation, n (%)	0 (0	113 (23.3)	**< 0.001**
ACEI/ARB, n (%)	6 (6.1)	198 (40.8)	**< 0.001**
Loop diuretics, n (%)	17 (34.7)	383 (79)	**< 0.001**
Beta-blocker, n (%)	7 (14.3)	291 (60)	**< 0.001**
Anticoagulants, n (%)	3 (6.1)	168 (34.6)	**< 0.001**
MRA, n (%)	8 (16.3)	212 (43.7)	**< 0.001**
LTOT, n (%)	3 (6.1)	68 (14)	**0.180**

The data are presented as median (IQR) or number (%)

ACEI, angiotensin-converting enzyme inhibitors; ARB, angiotensin receptor blockers; BMI, body mass index; DPAH, drug-induced pulmonary hypertension; HPAH, heritable pulmonary arterial hypertension; IPAH, idiopathic pulmonary arterial hypertension; LTOT, long-term oxygen therapy; MRA, mineralocorticoid receptor antagonist

### Comparison of invasive and non-invasive parameters in the group of patients with a positive (acute responders) and negative APV-test (non-acute responders)

Patients with a positive APV-test had a less advanced functional class than patients with a negative result (*p* < 0.001). Moreover, the population of patients with a positive APV-test was characterized by a longer distance walked in a 6-minute walking test and significantly lower values of NTproBNP, as compared to patients with a negative pulmonary vasoreactivity test (540 m/IQR:480–560/ vs. 280 m/IQR:160–390/, *p* < 0.001; 503.2 pg/ml/IQR:214.6–1533/ vs. 2019 pg/ml/IQR:806–4286/, *p* < 0.001; respectively). With regard to echocardiographic parameters, both groups (acute responders and non-acute responders) showed a statistically significant difference in the size of the right atrium (RAA) and TAPSE value (21 cm²/IQR:17–25/ vs. 25 cm²/IQR:20–31/; 19 mm/IQR:17–21/ vs. 17 mm/IQR:14–21/; respectively). Baseline hemodynamic parameters assessed during RHC did not differ significantly between groups with respect to PAP (mPAP and sPAP), PVR or CO; however, significant differences were observed in PCWP, RAP, and SvO₂ (PCWP: 7 mmHg [IQR 6–9] vs. 10 mmHg [IQR 7–12]; RAP: 5 mmHg [IQR 3–6] vs. 8 mmHg [IQR 5–11]; SvO₂: 73% [IQR 69–76.8] vs. 66% [IQR 59–72] in acute responders and non-acute responders, respectively), which may partially reflect differences in overall disease severity. No statistically significant differences were demonstrated in the assessment of selected ECG parameters in both study groups. Table 2 presents a comparison of invasive and non-invasive parameters in the groups of patients with a positive and negative APV-test.

**Table 2:** Comparison of invasive and non-invasive parameters in the groups of patients with a positive (acute-responders) and negative (non-acute responders) acute pulmonary vasoreactivity test

Disease characteristics at baseline	Acute responders *n* = 49	Non-acute responders *n* = 485	*p*-value
**Functional parameters**			
WHO-FC, n (%)			**< 0.001**
I	3 (6.1)	2 (0.4)	
II	17 (34.7)	76 (15.9)	
III	26 (53.1)	271 (56.6)	
IV	3 (6.1)	130 (27.1)	
6MWT [m], median (IQR)	540 (480–560)	280 (160–390)	**< 0.001**
NTproBNP [pg/ml], median (IQR)	503.2 (214.6–1533)	2019 (806–4286)	**< 0.001**
**Echocardiography**			
TAPSE [mm] (IQR)	19 (17–21)	17 (14–21)	**0.022**
RAA [cm²], median (IQR)	21 (17–25)	25 (20–31)	**< 0.001**
Pericardial effusion, n (%)	9 (18.4)	101 (21.0)	0.665
**Electrocardiography**			
Cardiac axis deviation, n (%)			0.438
Intermediate	25 (56.8)	216 (49.5)	
Right	18 (40.9)	192 (44.0)	
Left	1 (2.3)	28 (6.4)	
Intraventricular conduction disorders, n (%)			0.975
RBBB	3 (30.0)	48 (32.9)	
iRBBB	5 (50.0)	72 (49.3)	
LBBB	0 (0.0)	2 (0.44)	
iLBBB	0 (0.0)	3 (0.66)	
Non-specific	2 (20.0)	26 (17.8)	
**Right heart catheterization**			
sPAP [mmHg], median (IQR)	76 (63.5–83)	74 (59–92)	0.3491
mPAP [mmHg], median (IQR)	47(38–51)	46 (36–56)	0.5265
PCWP [mmHg], median (IQR)	7 (6–9)	10 (7–12)	**< 0.001**
SvO2 [%], median (IQR)	73 (69-76.8)	66 (59–72)	**< 0.001**
PVR [WU], median (IQR)	8.4 (6.5–11.5)	9.2 (5.8–13)	0.5953
CO [l/min], median (IQR)	4.31 (3.76–5.02)	3.92 (3.23–4.9)	0.1204
RAP [mmHg], median (IQR)	5 (3–6)	8 (5–11)	**< 0.001**

The data are presented as median (IQR) or number (%)

APV-test, acute pulmonary vasoreactivity test; CO, cardiac output; iLBBB, incomplete left bundle branch block; iRBBB, incomplete right bundle branch block; LBBB, left bundle branch block; mPAP, mean pulmonary arterial pressure; NTproBNP, N-terminal pro-B-type natriuretic peptide; PCWP, pulmonary wedge pressure; PVR, pulmonary vascular resistance; RAA, right atrial area; RAP, right atrial pressure; RBBB, right bundle branch block; sPAP, systolic pulmonary arterial pressure; SvO2, mixed venous oxygen saturation; TAPSE, tricuspid annular plane systolic excursion; WHO-FC, World Health Organization Functional Class, 6MWT, 6-minute walk test

### All-cause mortality

**Fig. 2:**
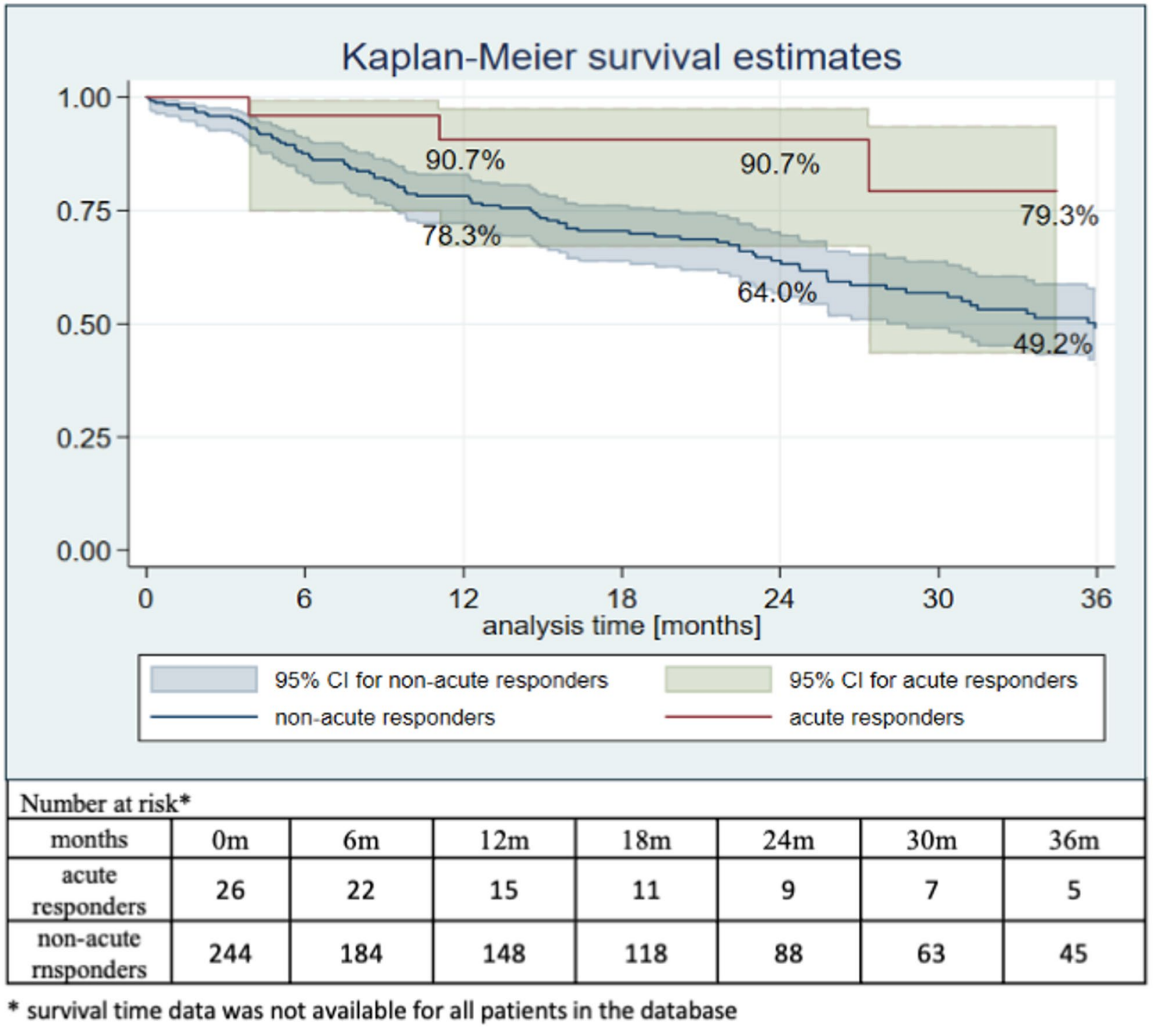
Kaplan–Meier survival curve in the groups of acute responders and non-acute responders (*p* = 0.039)

All-cause mortality was lower in patients with a positive APV-test (acute responders – 11.54%) compared with those with a negative APV-test (non-acute responders – 36.07%; *p* = 0.039) (Fig. 2). Survival curves demonstrate an early separation between groups; however, differences in follow-up duration and the progressive reduction in the number of patients at risk over time should be taken into account when interpreting these findings.

As this survival analysis is unadjusted, baseline differences between acute responders and non-responders, such as age, comorbidity burden, and functional status, may have contributed to the observed survival outcome.

**Fig. 3:**
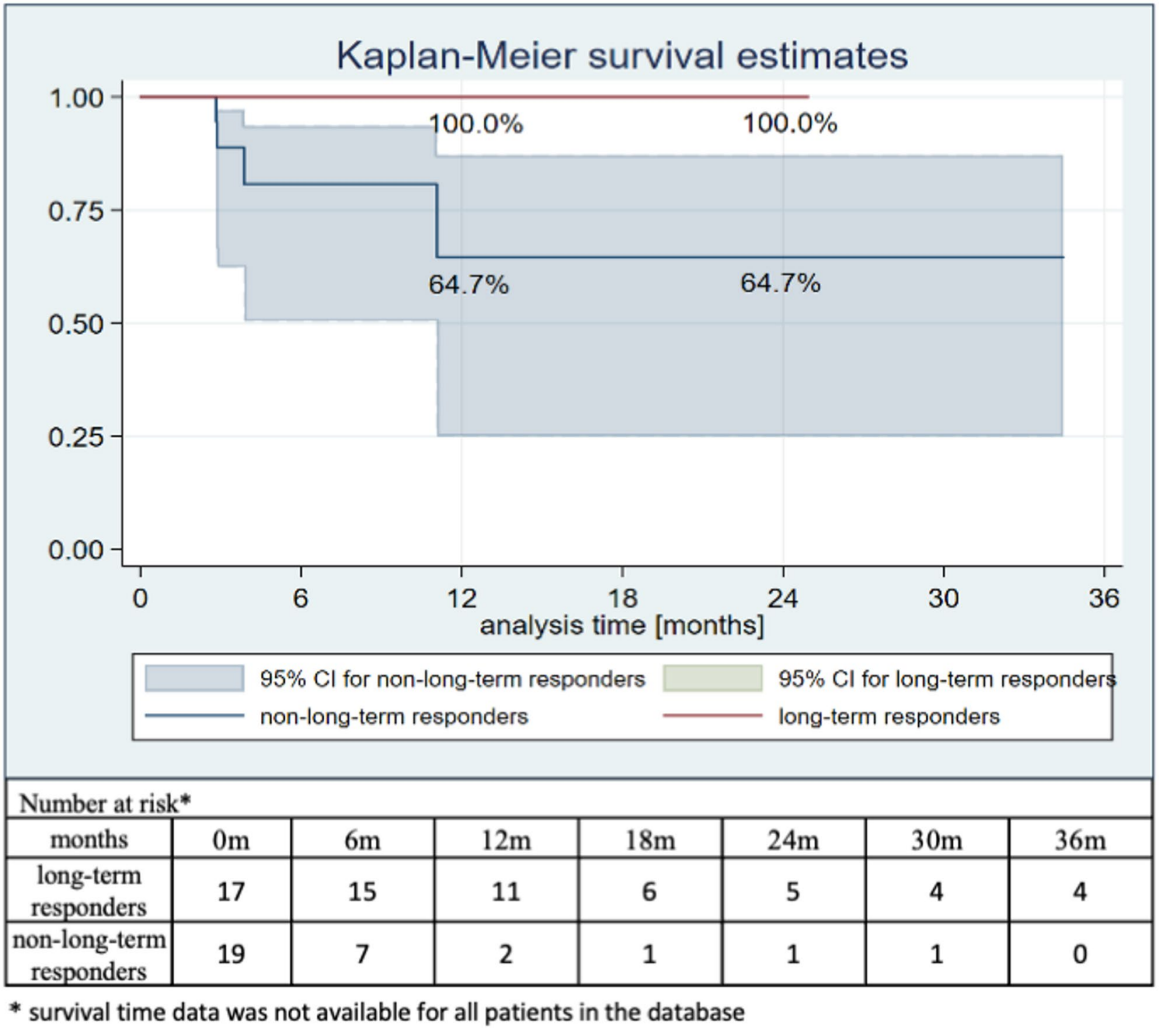
Kaplan–Meier survival curve in the groups of non-long-term responders and long-term responders (*p* = 0.05)

A long-term response to CCB therapy was observed in 55.1% (*n* = 27) of acute responders. The median follow-up for survival was longer in long-term responders than in non-long-term responders (14.7 months [IQR: 10.7–24.9] vs. 4.1 months [IQR: 3.0–11.1]). During the observation period, mortality events occurred more frequently in non-long-term responders than in long-term responders (21.05% vs. 0%; *p* = 0.05) (Fig. 3). Interpretation of survival patterns should consider the temporal distribution of events and the decreasing number of patients at risk, particularly in the non-long-term responder group.

## Discussion

**Fig. 4:**
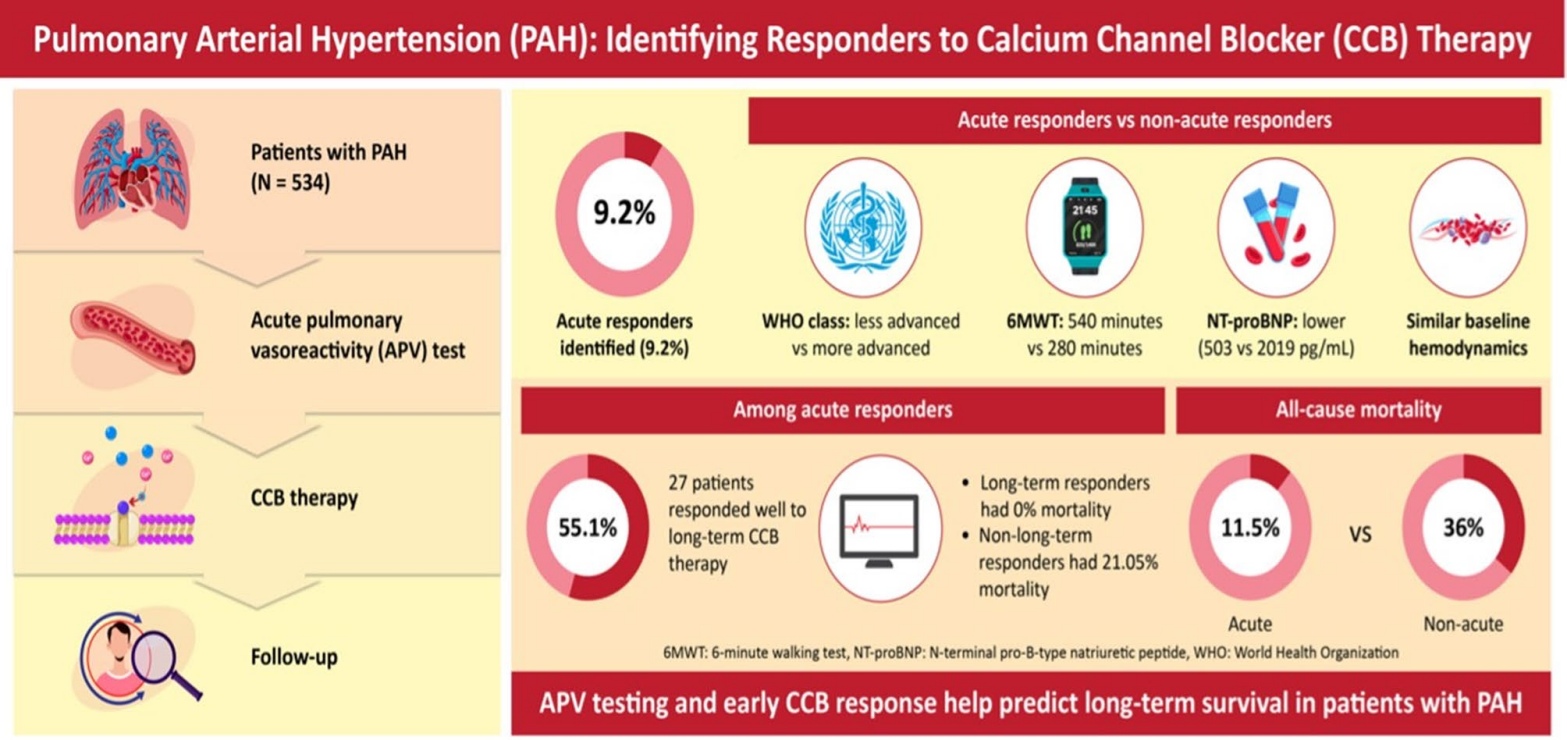
Illustration presenting a graphical representation of the results obtained

The present study analyzed data of a unique population of patients with PH from 21 Polish reference centers of diagnosis and treatment of PH accredited by the National Health Fund (Polish population – BNP-PL). The above subpopulation comprised patients with PH who had an APV-test done, and those who met the hemodynamic criterion of a so-called responder [2]. The above population was characterized with regard to coexisting diseases and a typical invasive and non-invasive assessment, followed by mortality assessment and demonstration which profile of patients treated chronically with a CCB had the best prognosis with respect to long-term survival (Fig. 4).

Three main conclusions resulting from the above study are as follows. Patients with PH and positive APV-test, as compared to those with a negative test, were characterized by better baseline non-invasive parameters (6MWT, NTproBNP level, WHO functional class, ECHO) and lower prevalence of coexisting diseases, while the differences in hemodynamics between these two groups were minor (PCWP, RAP, SvO2). All-cause mortality was lower in patients with a positive APV-test (acute responders) compared to those with a negative APV-test (non-acute responders). Similarly, survival was higher among patients with a persistent long-term response to CCB therapy (long-term responders) than in those requiring escalation of PH treatment (non-long-term responders). However, these survival analyses should be interpreted with caution, as follow-up duration differed substantially between the compared groups and the number of patients at risk decreased rapidly over time, particularly among non-long-term responders.

Most of all, it must be keep in mind that patients with PAH and positive PAV-test constitute a unique patient subpopulation, even in specialized centers, and data on this group of patients in world literature are scarce [3, 5–8, 16, 17]. Our study is exceptional in collecting and analyzing data from this unique patient group across Poland, although long-term responders were defined solely by clinical criteria—WHO functional class I/II and continued CCB monotherapy—due to limited hemodynamic follow-up data. While this approach allowed assessment of treatment response, it differs from current ESC/ERS guideline definitions that incorporate hemodynamic reassessment, which may lead to an overestimation of long-term responders and limits direct comparability with other studies.

The importance of the APV-test, regardless of the type of substance used to conduct the test, was already confirmed years ago [18–20]. However, it is generally known that some patients with a positive APV-test maintain their good hemodynamic response for years, and have a good prognosis, while others lose it very quickly and require escalation of the therapy targeted at pulmonary arterioles. Based on APV-test results and long-term assessment of patients receiving pharmacotherapy with a CCB, it is difficult to determine which group of patients will have better long-term results and very good long-term survival [5–7].

For several years, it has been also discussed how to define a patient with a good long-term hemodynamic response, i.e. if it can be done solely with the use of hemodynamic parameters, which has been recommended in the current guidelines of the European Society of Cardiology, or perhaps it can be done with the use of a specific stratification scale [2, 8].

In their latest study, Gerhardt et al. analyzed a very large group of patients with a positive APV-test from 15 reference centers of diagnosis and treatment of PH [8]. In the Polish registry, a positive acute hemodynamic response occurred in 9.2% patients, and in the above-mentioned registry in 9.7%, which is comparable to other data available in world literature, where the index is around 10% [5, 21].

In the above registry, similarly as in ours, a significant part of the population of patients with a positive APV-test and those who received Ca-blocker for a chronic therapy, were patients with IPAH (98.8% vs. 100%), and similarly, most of these patients were in WHO functional class II or III at the moment of diagnosis (25.2% vs. 34.7% - II WHO; 63.5% vs. 53.1% - III WHO, respectively). In our study group, NTproBNP level was slightly higher (503.2 pg/ml vs. 368 pg/ml, respectively), and the distance in the six-minute walking test (540 m vs. 416 m, respectively) was longer than in the study of Gerhardt et al. A hemodynamic profile of patients with a APV-test was almost identical in both registries (mPAP 47 mmHg vs. 47.1 mmHg; PCWP 7 mmHg vs. 8.1 mmHg; CO 4.3 l/min. vs. 4.5 l/min.; PVR 8.4 WU vs. 9.5 WU; Polish registry vs. Gergardt et al. registry, respectively). To define long-term responders in our Polish registry, we relied on simple clinical criteria (WHO functional class I/II and continued CCB monotherapy) due to limited availability of hemodynamic data, in contrast to other studies that use guideline-based definitions. Despite this limitation, conclusions from our study align with previous reports: patients with a positive APV-test have higher survival than those with a negative test. Similarly, the group of long-term responders, as reported by Gerhardt et al. (survival 98.5% vs. 73% for long-term vs. non-long-term responders), demonstrated markedly better survival than non-long-term responders, even when simple clinical criteria are applied.

Nevertheless, heterogeneity in follow-up duration and the rapid decline in the number of patients at risk over time warrant cautious interpretation of long-term survival estimates.

Overall, long-term responders exhibit very good long-term survival, consistent with findings from both earlier studies and our registry (including Sitbon et al.). However, confirmation in cohorts with more uniform and extended follow-up is needed to more precisely define long-term prognosis.

Table 3 compares the definitions and survival rates of acute and long-term responders across Sitbon et al., Gerhardt et al., and our study.

**Table 3:** Comparison of the definitions and survival rates of acute responders and long-term responders, in reference to our document, with the studies by Sitbon et al. and Gerhardt et al

Study	Definition of acute responder	Definition of long-term responder	1 year survival (%) acute/long-term responders	3yrs survival (%) acute/long-term responders	5yrs survival (%) acute/long-term responders
Sitbon et al. 2005	Fall in both mean pulmonary artery pressure (PAP) and pulmonary vascular resistance (PVR) > 20%	NYHA FC class I or II after at least 1 year on CCB monotherapy	-/97.4	-/97.4	-/97.4
Gerhardt et al. 2024	Decrease of mean pulmonary artery pressure by ≥ 10 mmHg to reach < 40 mmHg, without a decrease in cardiac output	Unchanged CCB therapy and WHO-FC I/II or low-risk status according to ESC/ERS risk assessment (3-strata), all at 12 months after initiation of CCB	95.9/100	90.4/100	88.3/98
Piłka et al. 2024	Decrease of mean pulmonary artery pressure by ≥ 10 mmHg to reach < 40 mmHg, without a decrease in cardiac output	WHO FC I/II and maintained monotherapy with Ca-blocker during the initial clinical assessment	90.7/100	79.3*/100*	-

* the observation was incomplete

Bearing that in mind, in the era of modern targeted therapy of pulmonary hypertension, the assessment of patients on Ca-blocker therapy should be applied,. The therapy should be escalated rapidly, by adding another drug targeted at pulmonary arterioles in patients who do not meet the criteria of a good long-term response to a chronic therapy with a CCB. A study of Kiani et al. demonstrated that the group of long-term-responders was not only in a lower functional class (NYHA I and II – 93.3%), but achieved a longer distance in a six-minute walking test and better hemodynamic parameters at baseline in comparison to patients without a good long-term response to treatment with Ca-blocker [24]. In regards to the group of long-term responders, the above study showed a significantly higher improvement in the distance covered in the six-minute walking test (437.43 ± 125.32 vs. 268.17 ± 130.06; *p* = 0.040), mixed venous oxygen saturation (71.84 ± 9.87 vs. 59.03 ± 9.95; *p* = 0.041), cardiac output, (4.76 ± 1.12 vs. 3.15 ± 0.90; *p* = 0.012), mean pulmonary arterial pressure (47.35 ± 12.70 vs. 67.23 ± 14.08; *p* = 0.034) and functional class (*p* = 0.001) as compared to the group where the effect of a good long-term response to treatment with CCB was not maintained [22].

Our analysis, similarly to previous studies, shows the group of acute responders are patients with a moderate disease severity, both in the initial invasive and non-invasive assessment, if compared to the group of non-acute responders [23–25]. There are also studies showing the value of a APV-test not only at the moment of making diagnosis of PAH, but also during a specific therapy. A follow-up study of vasoreactivity already during treatment may contribute to detection of subgroups of high risk that may require careful monitoring and referring the patient for lung transplantation at the right time, which was shown in the study of Ishii et al. [26]. The analysis of Kaplan-Meier survival showed significantly lower survival in the group with lower vasoreactivity, as compared to the group with maintained vasoreactivity (log-rank test, *p* = 0.016) [26]. Unfortunately, taking into account the nature of our data, we were not able to contribute to deepening of the significance and value of the above finding.

## Conclusions

Our findings, derived from the first large and distinct Polish PAH cohort, provide robust evidence that the initial APV-test–guided clinical assessment is not only diagnostically valuable but also prognostically decisive. At the same time, given differences in follow-up duration and the limited number of patients remaining under observation at later time points, particularly among non-long-term responders, conclusions regarding long-term survival should be interpreted with appropriate caution. Uniquely, our study demonstrates that in this specific population, early identification of patients with a positive APV test must be complemented by a rapid clinical evaluation following the initiation of CCB therapy. This independent step enables the precise recognition of individuals with excellent long-term survival potential, as well as the timely detection of those who require swift escalation of pulmonary hypertension therapy with additional agents — ultimately maximising the chances of improved survival in a patient group rarely represented in prior literature.

### Study limitations

Several limitations of this study should be acknowledged. First, hemodynamic data were not systematically available immediately after the pulmonary vasoreactivity test, precluding a detailed assessment of early hemodynamic response. Second, long-term response to calcium channel blocker therapy could not be evaluated according to the full hemodynamic criteria recommended in current ESC/ERS guidelines, as responder status was defined using a simplified clinical approach. Third, although high-dose calcium channel blocker therapy was used in patients with a positive acute vasoreactivity test, detailed data on individual drug selection and exact dosing were not available for all patients. Fourth, the timing of follow-up assessments and survival analyses varied between patients, and for some individuals the duration of follow-up was relatively short, which may have influenced outcome comparisons between responder groups. Finally, as this was a registry-based analysis from referral centers, patient selection, vasoreactivity testing practices, and treatment escalation strategies may have differed between sites, potentially introducing heterogeneity and limiting the generalizability of the findings.

## Data Availability

Data available from the authors upon request.
